# Comparative efficacy and safety of six non-ergot dopamine-receptor agonists in early Parkinson's disease: a systematic review and network meta-analysis

**DOI:** 10.3389/fneur.2023.1183823

**Published:** 2023-06-16

**Authors:** Xiang-Ting Chen, Qian Zhang, Fei-Fei Chen, Si-Yuan Wen, Chang-Qing Zhou

**Affiliations:** Department of Neurology, Bishan Hospital of Chongqing Medical University, Chongqing, China

**Keywords:** Parkinson's disease, network meta-analysis, dopamine-receptor agonists, ropinirole, piribedil, rotigotine, pramipexole

## Abstract

**Background:**

Non-ergot dopamine agonists (NEDAs) have been used as monotherapy or as an adjunctive therapy to levodopa for many years. Novel long-acting formulations of NEDAs including pramipexole extended-release (ER), ropinirole prolonged-release (PR), and rotigotine transdermal patch have been developed. However, there is no strong evidence that a given NEDA is more potent than another. We performed a systematic review and network meta-analysis to evaluate the efficacy, tolerability and safety of six commonly used NEDAs in early Parkinson's disease (PD).

**Methods:**

Six NEDAs including piribedil, rotigotine transdermal patch, pramipexole immediate-release (IR)/ER, and ropinirole IR/PR were investigated. The efficacy outcomes including Unified Parkinson's Disease Rating Scale activities in daily life (UPDRS-II), motor function (UPDRS-III), and their subtotal (UPDRS-II + III), tolerability and safety outcomes were analyzed.

**Results:**

A total of 20 RCTs (5,355 patients) were included in the current study. The result indicated that compared with placebo, all six investigated drugs had statistically significant differences in the improvement of UPDRS-II, UPDRS-III, and UPDRS-II + III (except ropinirole PR in UPDRS-II). There were no statistically significant differences between six NEDAs for the UPDRS-II and UPDRS-III. For UPDRS-II + III, the improvement of ropinirole IR/PR and piribedil were higher than that of rotigotine transdermal patch, and piribedil was higher than that of pramipexole IR. The surface under the cumulative ranking curve (SUCRA) indicated that piribedil resulted in best improvement in UPDRS-II and UPDRS-III (0.717 and 0.861, respectively). For UPDRS-II + III, piribedil and ropinirole PR exhibited similar improvement and both had high rates (0.858 and 0.878, respectively). Furthermore, piribedil performed better as monotherapy, ranking first in the improvement of UPDRS-II, III, and II + III (0.922, 0.960, and 0.941, separately). With regard to tolerability, there was a significant increase in overall withdrawals with pramipexole ER (0.937). In addition, the incidence of adverse reaction of ropinirole IR was relatively high (nausea: 0.678; somnolence: 0.752; dizziness: 0.758; fatigue: 0.890).

**Conclusions:**

In this systematic review and network meta-analysis of six NEDAs, piribedil exhibited better efficacy, especially as monotherapy, and ropinirole IR was associated with a higher incidence of adverse events in patients with early PD.

## 1. Introduction

Parkinson's disease (PD) is a neurodegenerative disorder that affects millions of people worldwide (1). The prevalence of PD is ~0.3% (2), the prevalence in older individuals over 65 years old is 1%−2%, and the prevalence in those over 85 years old is 3%−5% (3). PD has become a heavy burden for families and society.

Dopamine agonists (DAs) have been used as monotherapy for early PD or as an adjunctive therapy to levodopa for advanced PD for many years (4). Because of the potential for cardiac valve fibrosis or adverse retroperitoneal effects, ergot DAs are no longer used as first-line therapy in PD (5). Non-ergot dopamine agonists (NEDAs) continue to be first-line agents and novel long-acting formulations of NEDAs including pramipexole extended-release (ER), ropinirole prolonged-release (PR), and rotigotine transdermal patch have been developed. In comparison to three-times daily administration of standard NEDAs, once-daily administration of long-acting NEDAs provides a more stable plasma concentration and prolongs the duration of striatal dopamine receptors stimulation (6). Moreover, recent findings have demonstrated that once-daily administration of long-acting NEDAs may improve patients' adherence to treatment (7).

Over the past decades, many randomized controlled trials (RCTs) have been conducted to evaluate the efficacy and safety of NEDAs in early PD, but no head-to-head RCT have been conducted to evaluate all commonly used NEDAs. To our knowledge, there is no strong evidence that a given active NEDA is more potent than another. Furthermore, no network meta-analysis (NMA) has been performed to evaluate the long-acting and standard NEDAs, respectively (8–12). Nowadays, many types of medications including pramipexole ER, ropinirole PR, and rotigotine transdermal patch are available for symptomatic treatment, which is difficulty for clinicians to choose a DA for early PD patients. Previously, we have performed a NMA to compare six NEDAs as an adjunct to levodopa in advanced PD (13). NEDAs can also be used for early PD. Therefore, we performed a NMA to evaluate six commonly used NEDAs (rotigotine transdermal patch, ropinirole IR, ropinirole PR, pramipexole IR, pramipexole ER, piribedil) in early PD.

## 2. Methods

### 2.1. Search strategy

Our analysis followed the principles of the Preferred Reporting Items for Systematic Reviews and Meta-Analyses (PRISMA) extension statement (14). With the assistance of a medical librarian, a search strategy (Supplementary Appendix 1) was developed, and a systematic search of the medical literature was conducted using the MEDLINE (PubMed interface), EMBASE, and Cochrane Controlled Register of Controlled Trials databases. Articles published between January 1, 1996, and October 1, 2022 were retrieved in the primary search. The database-search strategy was sensitive and broad, utilizing a collection of search terms previously used in published systematic reviews of pharmacotherapy for PD. This article is based on previously conducted studies and does not contain any studies on human participants or animals performed by any of the authors. No ethical review was required for this publication.

### 2.2. Study selection

All titles and abstracts were screened by two reviewers (Xiang-Ting Chen and Qian Zhang) to determine eligibility, independently, for inclusion against our PICO criteria to improve the integrity of this study. Disagreement was resolved by discussion, and if no agreement could be met, an adjudicator (Chang-Qing Zhou) was included to resolve any disagreement.

#### 2.2.1. Inclusion criteria

(1) Participants: age: adults (2212≥18 years old); Race: any; Gender: any; Disease: early PD. (2) Interventions: rotigotine transdermal patch, ropinirole IR, ropinirole PR, pramipexole IR, pramipexole ER, piribedil. The above interventions can be used as monotherapy or in combination with levodopa. (3) Comparators: any comparator, including but not limited to: placebo, NEDAs. (4) Outcome measures. Efficacy outcomes: unified Parkinson's Disease Rating Scale-Activities of daily living (UPDRS-II) for activities of daily living, Unified Parkinson's Disease Rating Scale-Motor (UPDRS-III) for motor function, and Unified Parkinson's Disease Rating Scale—motor function and activities of daily living (UPDRS-II + III) scores. Tolerability outcomes: overall withdrawals, withdrawals due to adverse events (AEs), withdrawals due to lack of efficacy. Safety outcomes: the incidence of AEs (≥1 AE) or serious AE (SAE). Furthermore, we extracted all AEs and calculated the corresponding incidence of AEs. And statistical analysis of adverse reactions with high incidence. (5) Study design: randomized controlled trials (RCTs).

#### 2.2.2. Exclusion criteria

PD patients who receive surgical treatment; PD patients with mental disorders; PD patients with clinically relevant hepatic, renal, or cardiac disorders; studies with insufficient data; duplicated publications; system evaluation, or the summary article.

### 2.3. Data extraction

Data extraction was also conducted independently and in duplicate by two reviewers (Fei-Fei Chen and Si-Yuan Wen), with disagreement resolved through an adjudicator (Chang-Qing Zhou). Briefly, a number of variables pertaining to study design, interventions, patient characteristics, and outcomes were collected. The following information was extracted: the last name of the first author, year of publication, country, Hoehn and Yahr stage of subjects, number of subjects, time of follow-up (in weeks), age of subjects, the proportion of female subjects, duration of PD among the subjects (in years), agent dosage, disease severity at baseline, duration of treatment, and the proportion of patients receiving other medications. For the patient-level variables, data regarding the Hoehn and Yahr score were collected, and according to this scale, a score of < 3 indicates early PD. For a complete list of variables extracted, please see Supplementary Appendix 2.

### 2.4. Risk of bias assessment

The Cochrane Risk of Bias Tool for randomized clinical trials (RoB 2) (15) was used to assess the risk of bias in the RCTs. The RoB 2 tool is structured into five bias domains: randomization process, deviations from intended interventions, missing outcome data, measurement of the outcome, and selection of the reported result. Each domain was judged as either “Yes (low risk),” “No (high risk)” or “Unclear (uncertain risk).” These judgments led to grading the studies as “low risk of bias” when no aspects were defined as “unclear” o “no”; “some concerns” if at least one domain was deemed “unclear” but not “no” for any single domain; or “high risk of bias” when “no” was reached for at least one domain.

### 2.5. Statistical analysis

For each outcome of interest, analyses were performed on the change from baseline in the UPDRS-II, UPDRS-III, and UPDRS-II + III. Different intervention measures were compared using Bayesian treatment NMA depending on non-informative priors for effective sizes as well as precision, following the Bayesian models recommended (16). All Bayesian models were performed in R software (version 4.2.0) using the gemtc package and a Gibbs sampler for computing a Markov Chain Monte Carlo (MCMC) simulation. The number of simulation chains is four, and the number of tuning iteration and simulation iterations was 10,000 and 50,000, respectively.

All the considered efficacy outcomes were continuous, and for that reason, the employed effect measure in all Bayesian NMA models was the mean difference (MD) and estimation uncertainty was represented by the corresponding 95% credible interval (CrI), which is the difference between the mean change from baseline in two or three intervention groups. Taking tolerability and AEs into consideration, the effect measures used were odds ratio (OR) and 95% CrI for binary outcomes. As the intervention group standard deviations (SDs) were not reported in many of the identified trials, these data were either approximated from the reported statistics or imputed. When no variability measures were reported, imputation of the maximum SD from another study using the same measurement scale was performed. When no data were available to calculate the SD, the median SD (reported and approximated) of the other trial intervention groups was imputed. In addition, when studies did not report mean change, these values were calculated as the arithmetic difference between the baseline and follow-up (17).

Convergence was assessed using standard diagnostics. All approaches were used under the fixed-effects and random-effects framework, and model fit was assessed by comparing the deviance information criterion (DIC) of the fixed-effects and random-effects models. We evaluate model convergence by examining MCMC error, DIC, and plotting trace plot and density plot. Homogeneity and consistency assumptions were verified using node splitting (18). In addition, the surface under the cumulative ranking curve (SUCRA) was created to evaluate the ranking probabilities for different medications on various outcomes to select the best treatment option. Moreover, the consistency between direct and indirect evidence was assessed by the node-splitting method; a *P*-value < 0.05 was deemed inconsistent.

Furthermore, we re-analyzed NEDAs as monotherapy and combined levodopa treatment separately. As a sensitivity analysis, we undertook repeated analyses to evaluate the robustness of the model. Sensitivity analyses were also conducted using subgroups according to the following factors: excluding studies that applied imputation methods, and excluding studies with significant differences from baseline. Publication bias was assessed through a comparison-adjusted funnel plot.

## 3. Results

### 3.1. Baseline characteristics of the included literature

**Figure 1:**
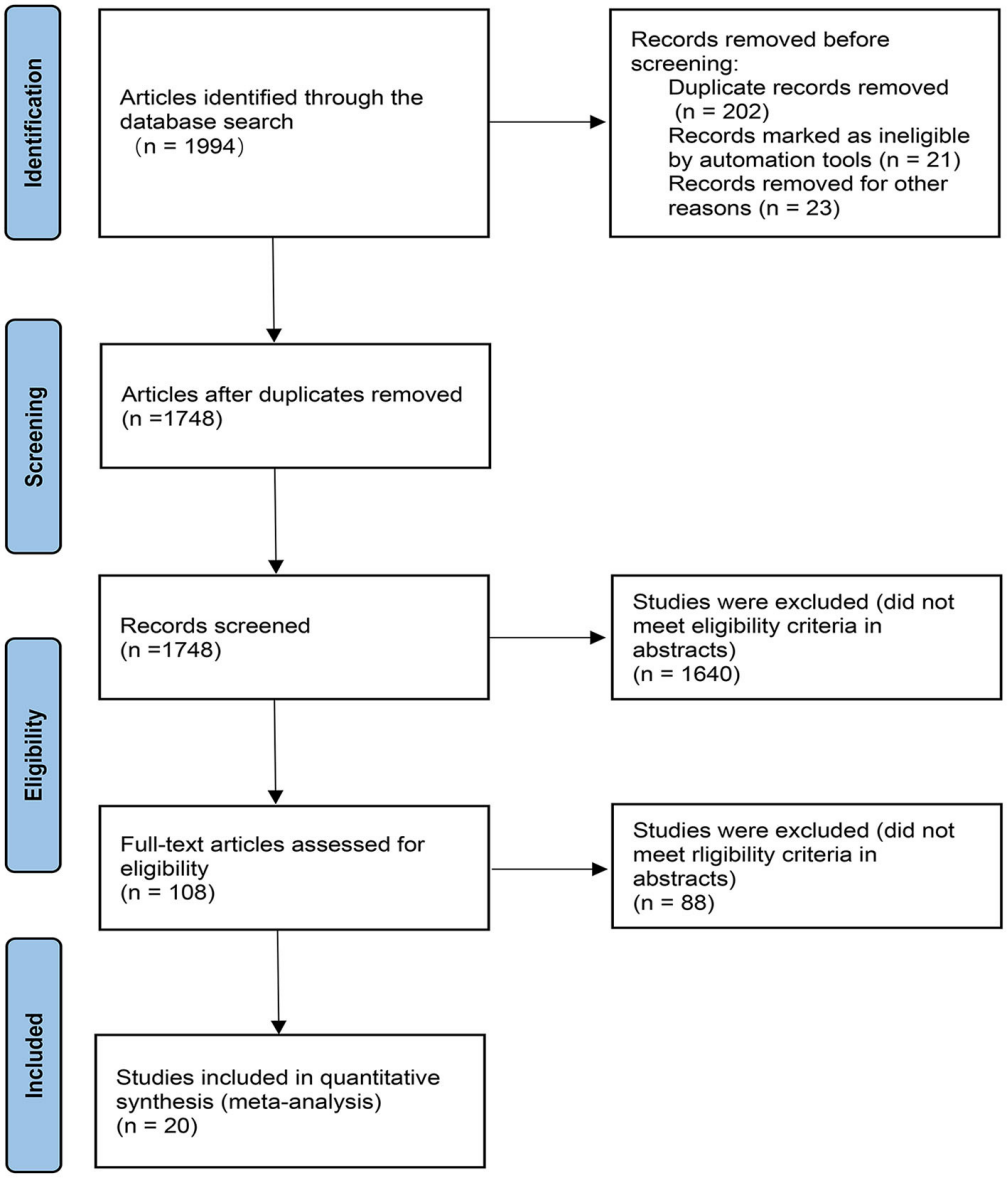
PRISMA flow chart. The flow chart shows the detailed procedures of the study screening and exclusion process. 20 studies were included in this network meta-analysis.

A total of 1994 records were retrieved, of which 20 RCTs (19–38) met the eligibility criteria and were included in the meta-analysis (Figure 1). The study included 5,355 early PD patients. Fifteen studies of these were NEDAs as monotherapy, and the other five were combined with levodopa. The sample size of the studies ranged from 60 to 561. In the 20 RCTs, 17 trials were two-arm trials and the remaining three trials were three-arm trials, facilitating indirect treatment comparisons between the six therapies. The baseline demographic and clinical characteristics of the included articles are shown in Table 1 [mean age of ~62 years and a lower proportion (around 41%) of women than men]. The follow-up period of our enrolled studies ranged from 9 to 96 weeks, with an average of 27 weeks. The mean disease duration was between 0.9 and 4.3 years. Trials were generally well-balanced with respect to patient baseline characteristics, and the Cochrane bias evaluation is shown in Supplementary Figures 1, 2. The potential scale reduction factors (PSRF) and trace plot and density plot indicating that the statistical analysis has achieved good convergence.

**Table 1:** The baseline characteristics of included studies in the network meta-analysis.

**References**	**Country**	**Comparison**	**Sample size**	**Age (years)**	**Women, No. (%)**	**Duration of PD (years)**	**Dosage**	**Follow-up (weeks)**	**Patient receiving other medications**	**Outcomes reported**
Adler et al. (20)	USA, et al. (25 sites)	C vs. A	241	62.9	37.8	2.0	0.75–24.0/–	24	Levodopa, MAO-B inhibitors	UPDRS-II + III, withdrawals, AEs
Kieburtz et al. (19)	USA. (20 centers)	E vs. A	282	61.7	35.1	1.9	1.5–6.0/–	11	Anticholinergics, amantadine, MAO-B inhibitors	UPDRS-II + III, withdrawals, AEs
Shannon et al. (21)	USA. et al. (18 sites)	E vs. A	335	62.7	39.4	1.8	0.375–4.5/–	24	MAO-B inhibitors	UPDRS-II, UPDRS-III, Withdrawals, AEs
Sethi et al. (22)	USA. et al. (22 sites)	C vs. A	241	61.9	–	1.9	3.0–24.0/–	24	Levodopa, MAO-B inhibitors	UPDRS-II + III, withdrawals, AEs
Blindauer et al. (23)	USA	B vs. A	242	61.3	36.4	1.3	4.5–18.0/–	11	Anticholinergics, amantadine, MAO-B inhibitors	UPDRS-II + III, withdrawals, AEs
Ziegler et al. (24)	France, Portugal (31 centers)	G vs. A	115	64.1	40.6	4.3	150/–	24	Levodopa	UPDRS-II, UPDRS-III, UPDRS-II + III, withdrawals, AEs
Rascol et al. (25)	Argentina, India, et al. (52 centers)	G vs. A	401	62.3	39.2	2.0	150–300/–	28	–	UPDRS-II, UPDRS-III, UPDRS-II + III, withdrawals, AEs
Thomas et al. (26)	USA	E vs. C	60	56.2	44.2	–	2.1–4.2/15.0–24.0	96	–	UPDRS-III, Withdrawals
Giladi et al. (27)	Israel	B vs. C vs. A	561	61.2	42.3	1.3	2.0–8.0/0.75–24.0/–	37	Anticholinergics, amantadine, MAO-B inhibitors	UPDRS-II, UPDRS-III, UPDRS-II + III, withdrawals, AEs
Jankovic e al. (28)	USA, Canada. et al. (50 sites)	B vs. A	277	62.9	34.6	1.3	4.5–13.5/–	24	Anticholinergics, amantadine, MAO-B inhibitors	UPDRS-II, UPDRS-III, UPDRS-II + III, withdrawals, AEs
Singer et al. (29)	USA, Mexico, et sl. (101 centers)	C vs. A	410	65.1	38.0	1.3	0.75–24.0/–	40	Anticholinergics, amantadine, MAO-B inhibitors	UPDRS-II, UPDRS-II + III, withdrawals, AEs
Stocchi et al. (30)	Belgium, the Czech, et al. (30 centers)	D vs. C	150	60.3	45.6	2.7	2.0–24.0/0.75–24.0	20	Anticholinergics, amantadine, MAO-B inhibitors	UPDRS-II, UPDRS-III, UPDRS-II + III, AEs
Barone et al. (31)	South Africa, et al. (76 centers)	E vs. A	296	67.0	52.7	4.0	0.125–1.0/–	12	Levodopa, anticholinergics, amantadine, MAO-B inhibitors	UPDRS-II, UPDRS-III, UPDRS-II + III, withdrawals, AEs
Hauser et al. (32)	USA, et al. (94 centers)	F vs. E vs. A	259	62.1	44.4	1.0	0.375–4.5/0.375–4.5/–	18	Anticholinergics, amantadine, MAO-B inhibitors	UPDRS-II, UPDRS-III, UPDRS-II + III, withdrawals, AEs
Rascol et al. (33)	France, Germany, et al. (26 centers)	F vs. E	156	63.7	43.6	3.3	1.5–4.5/1.5–4.5	9	Levodopa, anticholinergics, amantadine, MAO-B inhibitors	UPDRS-II, UPDRS-III, UPDRS-II + III, withdrawals, AEs
Kieburtz et al. (34)	Rochester, New York (39 centers)	E vs. A	157	62.7	26.8	2.6	1.5–2.25/–	12	Anticholinergics, amantadine, MAO-B inhibitors	UPDRS-II + III, withdrawals, AEs
Poewe et al. (35)	Argentina, Austriam, et al. (94 centers)	F vs. E vs. A	523	61.6	44.5	1.0	0.375–4.5/0.375–4.5/–	33	Anticholinergics, amantadine, MAO-B inhibitors	UPDRS-II, UPDRS-III, UPDRS-II + III, withdrawals, AEs
Sampaio et al. (36)	Australia, Czech Republic, et al. (78 centers)	E vs. A	226	61.8	41.6	0.9	1.5–4.5/–	24	Anticholinergics, amantadine, MAO-B inhibitors	UPDRS-II, UPDRS-III, UPDRS-II + III, Withdrawals, AEs
Mizuno et al. (37)	Japan (41 centers)	B vs. A	176	–	60.2	1.9	2.0–16.0/–	12	–	UPDRS-II + III, withdrawals, AEs
Zhang et al. (38)	China	B vs. A	247	59.4	39.3	1.0	2.0–8.0/–	12	Anticholinergics, amantadine, MAO-B inhibitors	UPDRS-II, UPDRS-III, UPDRS-II + III, withdrawals, AEs

A, Placebo; B, Rotigotine transdermal patch; C, Ropinirole Immediate Release (IR); D, Ropinirole Prolonged Release (PR); E, Pramipexole IR; F, Pramipexole Extended Release (ER); G, Piribedil; MAO-B Inhibitors, monoamine oxidase B Inhibitors; UPDRS-II, Unified Parkinson's Disease Rating Scale-activities of daily living; UPDRS-III, Unified Parkinson's Disease Rating Scale-Motor; UPDRS-II + III, Unified Parkinson's Disease Rating Scale—motor and activities of daily living.

### 3.2. Evidence of a network relationship

**Figure 2:**
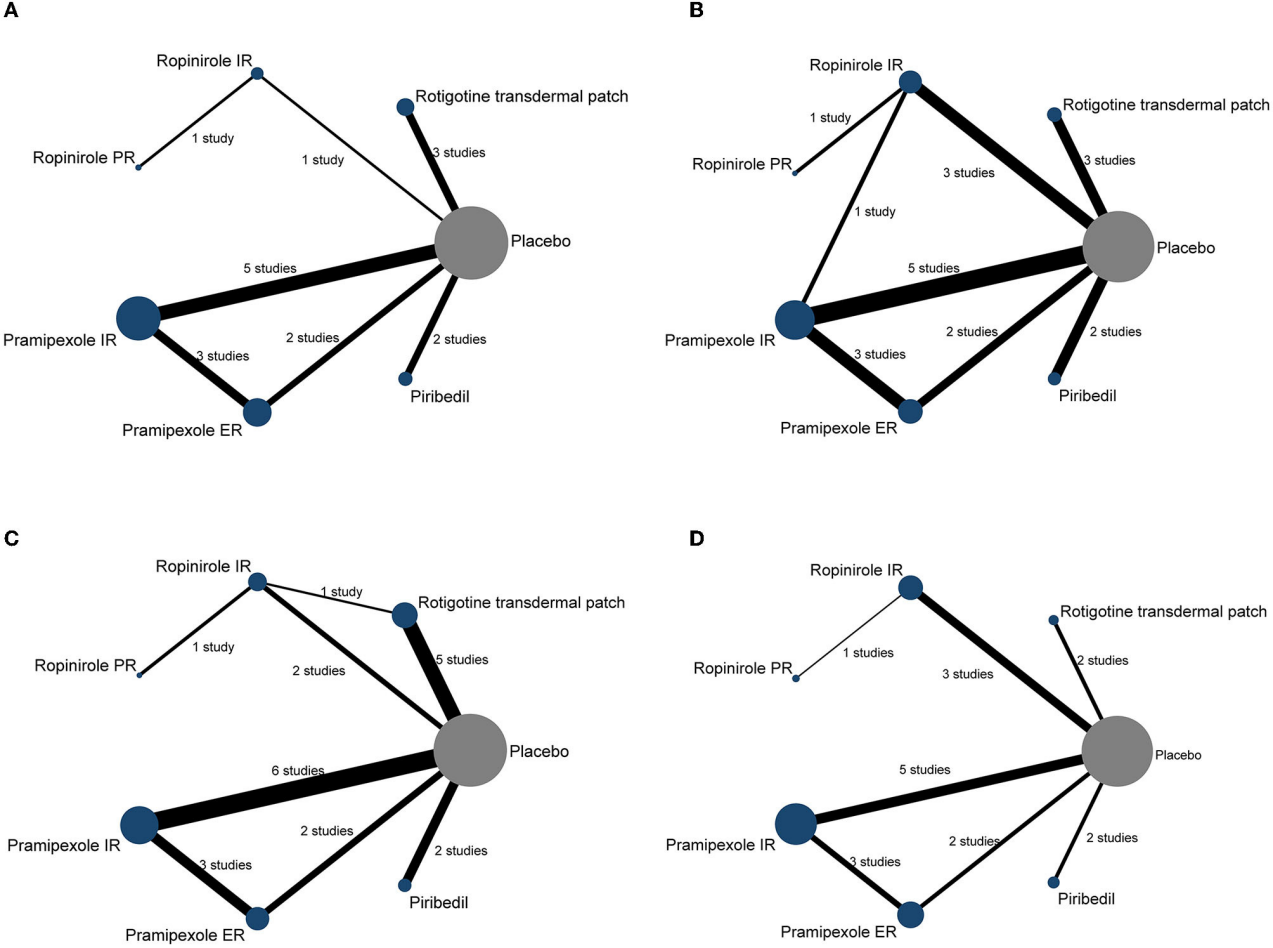
The network plot of included trials. Each node represents a therapy of PD. The nodes are weighted by the number of studies; the width of the edges is weighted by the standard errors; the solid line represents the direct comparison between the two interventions; the number between two nodes represents the number of studies involved in the head-to-head comparison. **(A)** UPDRS-II, **(B)** UPDRS-III, **(C)** UPDRS-II + III, and **(D)** the incidences of AEs (≥1 AEs).

All analyses were conducted by comparing each intervention (rotigotine transdermal patch, ropinirole IR, ropinirole PR, pramipexole IR, pramipexole ER, piribedil), respectively. To clarify the comparisons included in the NMA, a network plot was generated (Figure 2). Numbers in the circles illustrate the number of subjects. The width of the line is proportional to the total standard errors of the studies included. The contribution plots show the percentage of statistical contribution coming from direct and indirect evidence for each direct comparison in the network (Supplementary Figure 3).

### 3.3. Main results of the efficacy

The NMA was performed to promote result validity by merging direct and indirect evidence. Thirteen studies were included in the analysis of the UPDRS-II score, 13 studies for the UPDRS-III score, and 16 studies for the UPDRS-II + III score. Corresponding results are presented in Table 2.

**Table 2:** Network meta-analysis results according to UPDRS-II, UPDRS-III and UPDRS-II + III represented by MD and 95% CrI.

	**Placebo**	**Rotigotine transdermal patch**	**Ropinirole IR**	**Ropinirole PR**	**Pramipexole IR**	**Pramipexole ER**	**Piribedil**
**UPDRS-II (13 studies, 3,685 patients)**							
Rank	7	4	6	3	2	5	**1**
SUCRA	0.013	0.564	0.513	0.565	0.594	0.534	**0.717**
Rotigotine transdermal patch	**−1.62 (−2.44**, **−0.77)**						
Ropinirole IR	**−1.52 (−2.95**, **−0.11)**	0.10 (−1.56, 1.72)					
Ropinirole PR	−1.62 (−3.65, 0.42)	0.01 (−2.21, 2.16)	−0.09 (−1.55, 1.37)				
Pramipexole IR	**−1.66 (−2.29**, **−1.00)**	−0.04 (−1.08, 0.99)	−0.14 (−1.69, 1.43)	−0.04 (−2.17, 2.11)			
Pramipexole ER	**−1.58 (−2.46**, **−0.70)**	0.04 (−1.20, 1.22)	−0.06 (−1.75, 1.60)	0.04 (−2.20, 2.22)	0.08 (−0.75, 0.87)		
Piribedil	**−1.90 (−2.95**, **−0.68)**	−0.28 (−1.57, 1.18)	−0.39 (−2.08, 1.55)	−0.29 (−2.51, 2.18)	−0.25 (−1.45, 1.14)	−0.33 (−1.64, 1.23)	
**UPDRS-III (16 studies, 4,219 patients)**							
Rank	7	6	3	2	5	4	**1**
SUCRA	0.001	0.262	0.590	0.832	0.437	0.518	**0.861**
Rotigotine transdermal patch	**−3.39 (−5.08**, **−1.71)**						
Ropinirole IR	**−4.77 (−6.33**, **−3.21)**	−1.38 (−3.68, 0.92)					
Ropinirole PR	**−6.26 (−9.64**, **−2.87)**	−2.87 (−6.64, 0.93)	−1.48 (−4.48, 1.53)				
Pramipexole IR	**−4.23 (−5.42**, **−2.96)**	−0.82 (−2.87, 1.25)	0.56 (−1.27, 2.35)	2.03 (−1.43, 5.58)			
Pramipexole ER	**−4.46 (−6.33**, **−2.70)**	−1.07 (−3.56, 1.35)	0.31 (−2.04, 2.56)	1.79 (−1.97, 5.52)	−0.23 (−1.97, 1.39)		
Piribedil	**−6.33 (−8.61**, **−3.73)**	−2.94 (−5.72, 0.19)	−1.55 (−4.26, 1.51)	−0.08 (−4.11, 4.22)	−2.11 (−4.68, 0.71)	−1.86 (−4.71, 1.34)	
**UPDRS-II** + **III (16 studies, 4,417 patients)**							
Rank	7	6	3	**1**	5	4	2
SUCRA	0.001	0.241	0.713	**0.878**	0.358	0.451	0.858
Rotigotine transdermal patch	**−4.27 (−5.68**, **−2.92)**						
Ropinirole IR	**−7.21 (−9.17**, **−5.25)**	**−2.92 (−5.00**, **−0.83)**					
Ropinirole PR	**−8.82 (−12.84**, **−4.89)**	**−4.55 (−8.64**, **−0.48)**	−1.6 (−5.17, 1.86)				
Pramipexole IR	**−4.94 (−6.24**, **−3.58)**	−0.65 (−2.48, 1.27)	2.3 (−0.07, 4.64)	3.9 (−0.28, 8.17)			
Pramipexole ER	**−5.34 (−7.36**, **−3.45)**	−1.07 (−3.52, 1.31)	1.86 (−0.94, 4.6)	3.46 (−0.93, 7.93)	−0.43 (−2.28, 1.35)		
Piribedil	**−8.48 (−11.25**, **−5.52)**	**−4.22 (−7.27**, **−0.84)**	−1.26 (−4.58, 2.32)	0.31 (−4.45, 5.37)	**−3.56 (−6.55**, **−0.32)**	−3.12 (−6.44, 0.53)	

Values in bold represent statistically significant results. Bold font of SUCRA value indicates the highest SUCRA value, which can be interpreted as the estimated proportion of treatments better than the treatment in question. MD, mean difference; 95% CrI, 95% credibility interval.

The main results of the NMA showed that, using the UPDRS-II, rotigotine transdermal patch, ropinirole IR, pramipexole IR/ER, and piribedil demonstrated better efficacy than placebo (MD −1.62, 95% CrI −2.44 to −0.77; MD −1.52, 95% CrI −2.95 to −0.11; MD −1.66, 95% CrI −2.29 to −1.00; MD −1.58, 95% CrI −2.46 to −0.70; MD −1.90, 95% CrI −2.95 to −0.68, respectively). There were no statistically significant differences between ropinirole PR and placebo in the UPDRS-II.

Compared with placebo, rotigotine transdermal patch, ropinirole IR/PR, pramipexole IR/ER and piribedil, exhibited increased efficacy (MD −3.39, 95% CrI −5.08 to −1.71; MD −4.77, 95% CrI −6.33 to −3.21; MD −6.26, 95% CrI −9.64 to −2.87; MD −4.23, 95% CrI −5.42 to −2.96; MD −4.46, 95% CrI −6.33 to −2.70; MD −6.33, 95% CrI −8.61 to −3.73, respectively) in the UPDRS-III. The results of this analysis indicated that there were no statistically significant differences between six NEDAs for the UPDRS-II and UPDRS-III.

**Figure 3:**
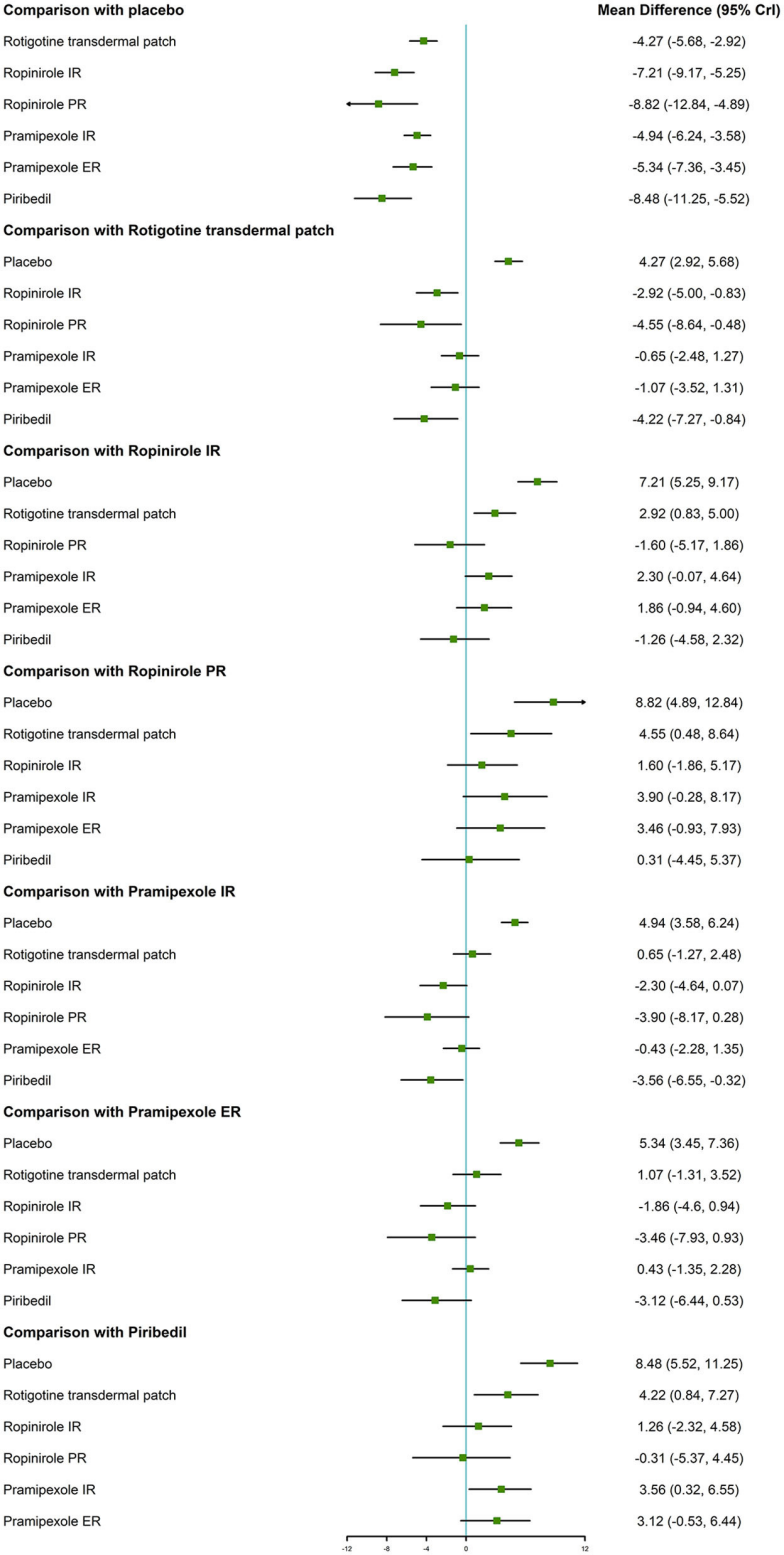
Forest plot for UPDRS-II + III.

With regard to the UPDRS-II + III, improvement rate was much higher in patients who took the six NEDAs compared to those who took the placebo. Moreover, the improvement rate with ropinirole IR/PR and piribedil was higher than that with rotigotine transdermal patch (MD −2.92, 95% CrI −5.00 to −0.83; MD −4.55, 95% CrI −8.64 to −0.48; MD −4.22, 95% CrI −7.27 to −0.84, respectively, Figure 3).

### 3.4. Tolerability outcomes

Compared to placebo, pramipexole IR and ER were associated with a higher incidence of overall withdrawals (OR 1.66, 95% CrI 1.18–2.45; OR 2.18, 95% CrI 1.35–3.70, Supplementary Table 1). Compared with ropinirole IR, pramipexole IR/ER and piribedil were associated with a higher incidence of overall withdrawals (OR 2.08, 95% CrI 1.27–3.51; OR 2.73, 95% CrI 1.50–5.13; OR 2.02, 95% CrI 1.03–3.85). Compared to rotigotine transdermal patch, pramipexole ER was associated with a higher incidence of overall withdrawals (OR 1.96, 95% CrI 1.08–3.64). With regard to withdrawals due to AEs, rotigotine transdermal patch, ropinirole IR and pramipexole IR/ER were associated with a higher incidence than placebo (OR 2.71, 95% CrI 1.45–4.97; OR 2.50, 95% CrI 1.50–4.11; OR 2.23, 95% CrI 1.39–4.06; OR 2.71, 95% CrI 1.25–6.35). Furthermore, rotigotine transdermal patch, ropinirole IR and pramipexole IR exhibited a lower risk of withdrawals due to lack of efficacy compared with placebo (OR 0.36, 95% CrI 0.15–0.86; OR 0.24, 95% CrI 0.12–0.48, OR 0.22, 95% CrI 0.08–0.53). There were fewer data for ropinirole PR, thus we were unable to assess the incidence rate of withdrawals for ropinirole PR.

### 3.5. Safety outcomes

The assessment of safety demonstrated that all six interventions resulted in significant increases in AEs compared with placebo (Supplementary Table 2). Compared with placebo, pramipexole IR and piribedil were associated with an increased risk of SAEs (OR 2.04, 95% CrI 1.05–4.46; OR 5.37, 95% CrI 1.88–16.06). We analyzed the incidence of nausea, somnolence, dizziness, headache, constipation, and fatigue (Supplementary Table 3). The results showed that rotigotine transdermal patch, ropinirole IR, and pramipexole IR/ER were associated with a higher incidence of nausea and somnolence than placebo. Additionally, ropinirole IR was associated with a significantly higher risk of dizziness and fatigue compared with placebo (OR 2.21, 95% CrI 1.34–3.57; OR 5.52, 95% CrI 1.24–29.97). Rotigotine transdermal patch and pramipexole IR were associated with a higher incidence of insomnia compared with placebo (OR 2.04, 95% CrI 1.01–4.31; OR 2.06, 95% CrI 1.11–3.78). Pramipexole IR/ER were associated with a higher incidence of constipation compared with placebo (OR 3.64, 95% CrI 2.12–6.92; OR 4.42, 95% CrI 2.11–10.69). There was no statistically significant difference in the incidence of headache in all the investigated drugs.

### 3.6. Cumulative ranking probability as a ranking scheme

**Figure 4:**
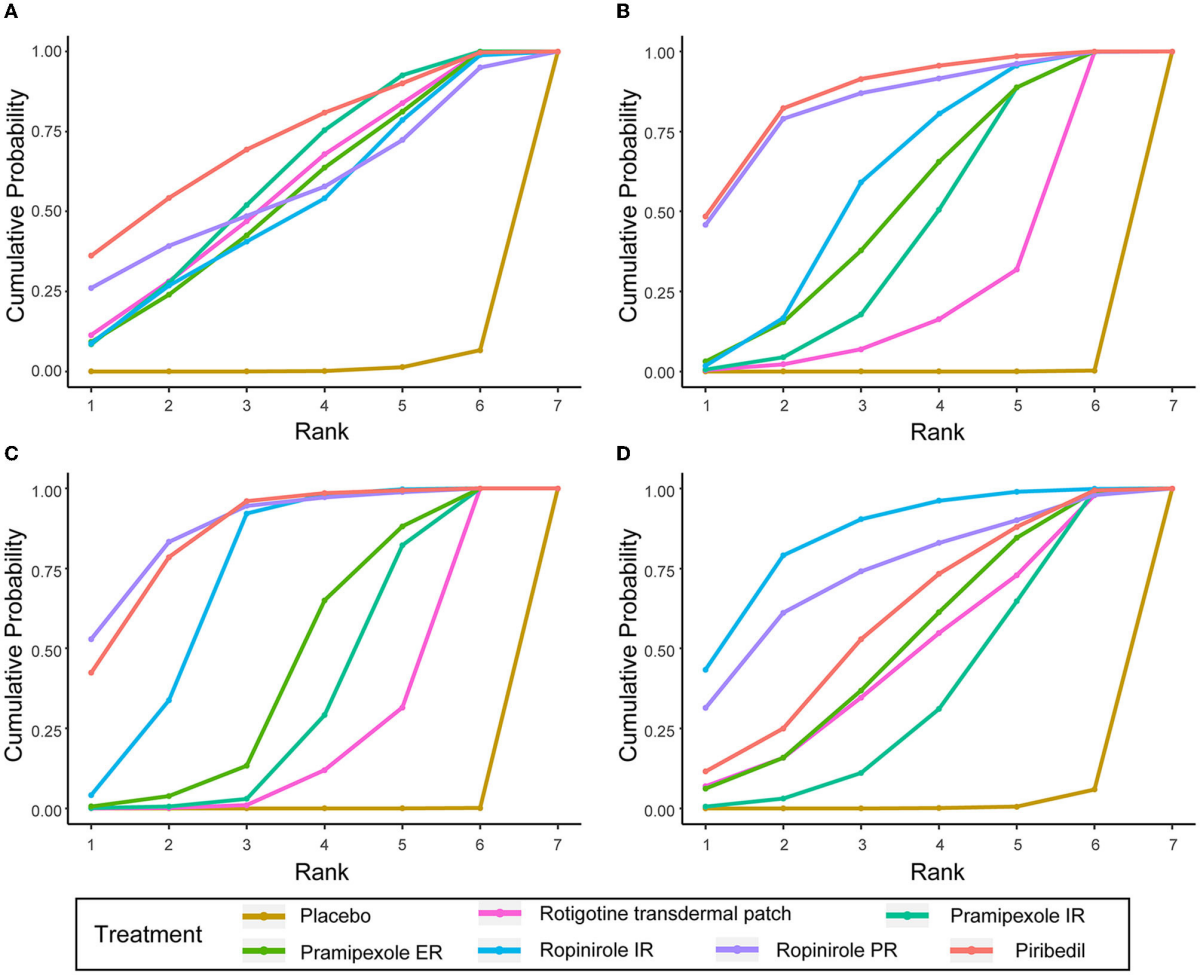
SUCRA of UPDRS-II, UPDRS-III, UPDRS-II + III, and ≥ 1 AEs. **(A)** UPDRS-II, **(B)** UPDRS-III, **(C)** UPDRS-II + III, **(D)** the incidences of AEs (≥1 AEs).

To better understand the results, the SUCRA values were calculated to assess the ranking probabilities of all medications on the investigated outcomes (Figure 4). As suggested by the ranking probabilities (Table 2), piribedil was the most effective medication in terms of the UPDRS-II in early PD (0.717), while the other five interventions had similar SUCRA values. With regard to improvement in the UPDRS-III, piribedil also ranked highest (0.861) followed by ropinirole PR (0.832) and ropinirole IR (0.590). With regard to the UPDRS-II + III, ropinirole PR and piribedil performed best (0.878 and 0.858, respectively).

In terms of tolerability (Supplementary Table 1), the tolerability of ropinirole PR could not be assessed due to a lack of data. Pramipexole ER ranked first in overall withdrawals (0.937). Rotigotine transdermal patch ranked first in withdrawals due to AEs (0.681) and ranked first in withdrawals due to lack of efficacy except for placebo (0.553). In terms of safety, patients taking ropinirole IR/PR were more likely to suffer from AEs (0.847 and 0.730, respectively, for ≥1 AE). Table 3 shows the SUCRA values of seven AEs. With regard to nausea, somnolence, dizziness and fatigue, ropinirole IR had the highest SUCRA value (0.678, 0.752, 0.758, and 0.890, respectively). As for headache and insomnia, rotigotine transdermal patch exhibited the highest SUCRA value (0.750 and 0.658, respectively). In terms of constipation, pramipexole ER achieved the highest SUCRA value (0.890).

**Table 3:** SUCRA of seven adverse events.

**Treatment**	**Nausea (19.8%)**	**Somnolence (16.8%)**	**Dizziness (11.9%)**	**Headache (9.1%)**	**Fatigue (7.7%)**	**Insomnia (7.3%)**	**Constipation (7.2%)**
Placebo	0.015	0.053	0.058	0.448	0.139	0.062	0.098
Rotigotine transdermal patch	0.629	0.488	0.469	**0.750**	0.691	**0.658**	0.282
Ropinirole IR	**0.678**	**0.752**	**0.758**	0.468	**0.890**	0.425	0.411
Ropinirole PR	0.610	0.547	0.704	0.520	0.579	–	0.456
Pramipexole IR	0.461	0.561	0.448	0.450	0.310	0.660	0.790
Pramipexole ER	0.467	0.656	0.494	0.368	0.391	–	**0.890**
Piribedil	0.639	0.447	0.569	–	–	0.695	0.576

Bold font indicates the highest SUCRA value, which can be interpreted as the estimated proportion of treatments worse than the treatment in question. The pooled incidence of AEs is indicated in parentheses.

SUCRA, surface under the cumulative ranking curves.

**Figure 5:**
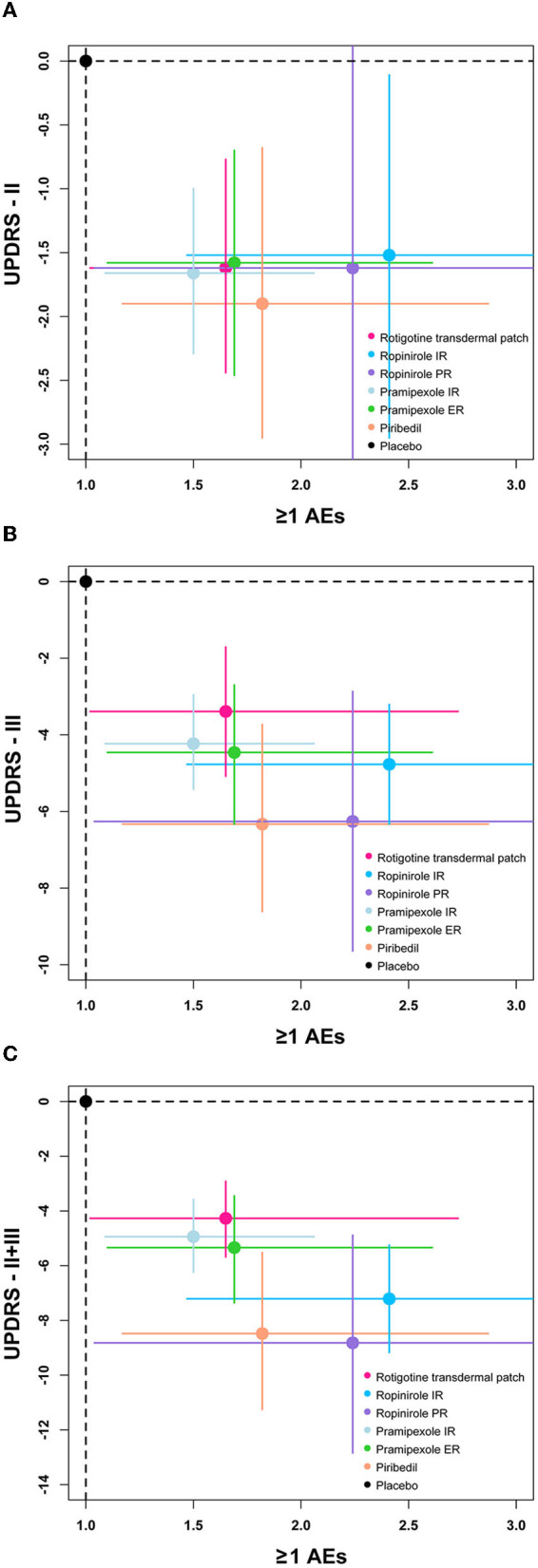
The clustered ranking plot of the network. Each plot shows MD (95%Crl) and OR (95%CrI) for two outcomes. Treatments lying in the lower-left corner are more effective and safer than the other treatments. OR, odds ratio. **(A)** Two-dimension plot of UPDRS-II and ≥1 AEs, **(B)** Two-dimension plot of UPDRS-III and ≥1 AEs, **(C)** Two-dimension plot of UPDRS-II + III and ≥1 AEs.

A clustered ranking plot combined with efficacy outcomes and ≥1 AE was also generated and the NMA results are presented visually in Figure 5.

### 3.7. As monotherapy and as an adjunct treatment to levodopa

We analyzed separately NEDAs as monotherapy and as an adjunct to levodopa for the treatment of early PD (Supplementary Table 4). We found that piribedil performed better as monotherapy, ranking first in the improvement of UPDRS-II, III, and II + III outcomes (0.922, 0.960, and 0.941, separately). These outcomes were largely consistent with results of our overall analysis. In contrast, pramipexole ER performed better than other NEDAs when used as an adjunct to levodopa, ranking first in UPDRS-II and II + III (0.738, 0.702) and second in UPDRS-III (0.606). Ropinirole IR was more effective as an adjunct to levodopa for early PD motor symptoms, with a statistically significant difference compared to placebo, and ranked first for improvement in UPDRS-III (0.827). For tolerability and safety analyses, the results for monotherapy or as an adjunct to levodopa were approximately the same as those of the primary analysis.

### 3.8. Assessment of heterogeneity, inconsistency and funnel plots

In the node-splitting plot (Supplementary Figure 4), the *P*-values were higher than 0.05, which indicated a relatively satisfactory consistency between direct and indirect evidence. The net heat plot suggested the source of inconsistency (Supplementary Figure 5).

The sensitivity analyses were almost consistent with the results of the main analysis. In the study by Thomas et al. (26) the treatment period was 96 weeks. When this study was excluded, the results were consistent with the results for all studies combined. Furthermore, when the analyses were limited to blinded studies, the results were unchanged.

The comparison-adjusted funnel plots did not reveal evidence of asymmetry (Supplementary Figure 6). The results of Begg and Egger's indicated no significant evidence of publication bias.

## 4. Discussion

To our knowledge, there is no strong evidence that a given active NEDA is more potent than another. This Bayesian NMA of RCTs evaluated the efficacy, tolerability, and safety of six commonly used long-acting and standard NEDAs, respectively, in early PD and provided evidence for clinicians to manage early PD patients.

In terms of efficacy, the six NEDAs resulted in a significant reduction in the UPDRS-II (except ropinirole PR), III, and II + III scores compared with placebo. More importantly, the aim of the current NMA was to quantitatively compare the six NEDAs, and sort comparisons according to various indices to choose the best treatment plan for early PD. The SUCRA values showed that piribedil was the best of the above drugs in managing symptoms of early PD, and ranked first in the UPDRS-II and UPDRS-III, and ranked second only to ropinirole PR in the UPDRS-II + III. Although a previous NMA (10) did not differentiate between long-acting and standard NEDAs, the results showed that piribedil was associated with a better ranking than rotigotine transdermal patch, pramipexole and ropinirole in terms of the UPDRS-II and UPDRS-III, which were consistent with the current results. Another NMA (9) found that there was no significant difference between pramipexole and piribedil in the UPDRS II/III scores in early PD, but it did not provide SUCRA values for comparison. Three NMAs (8, 11, 12) also proposed that pramipexole and ropinirole exhibited similar efficacy in PD; however, piribedil was not included. In addition, the current NMA showed that there was no significant difference between ropinirole PR and placebo in terms of the UPDRS II. Previous NMA (12) also showed that ropinirole did not improve the UPDRS II compared with placebo in early PD. Although other NMAs (8, 10–12) and meta-analyses (39) showed that ropinirole was associated with a significant improvement in the UPDRS-II compared with placebo, early PD and advanced PD were not analyzed separately, and ropinirole PR and ropinirole IR were not analyzed separately. Therefore, more research is needed to confirm the efficacy of ropinirole PR in early PD.

Furthermore, we analyzed separately NEDAs as monotherapy and as adjunct therapy with levodopa. We found that piribedil was more effective as monotherapy than other NEDAs in terms of efficacy, ranking first in improving UPDRS-II, III, and II + III. These results were broadly similar to those of the primary analysis. Pramipexole ER and ropinirole IR appeared to be more effective as adjunct therapy with levodopa. However, there were relatively few studies as adjunct therapy with levodopa in early PD. Most differences were not statistically significant, and SUCRAs are close. There are interventions, such as rotigotine transdermal patch and ropinirole ER, that have not been analyzed as adjunct therapy with levodopa because of the lack of RCT studies. Therefore, the results need to be interpreted with more caution.

Pramipexole ER was associated with a higher risk of overall withdrawals and the incidence of withdrawals due to AEs was only lower than rotigotine transdermal patch. Therefore, the high incidence of withdrawals may have caused the incidence of AEs due to pramipexole ER to be underestimated. Rotigotine transdermal patch was associated with a higher risk of withdrawal due to AEs than the other NEDAs. These results were similar to a previous meta-analysis of rotigotine transdermal patch in early PD (40).

In terms of safety, all six NEDAs showed similar AEs in early PD, and both nausea and somnolence were the most common AEs. Ropinirole IR/PR were associated with a higher risk of AEs than the other NEDAs. Furthermore, ropinirole IR demonstrated a significantly higher incidence of nausea, somnolence and dizziness. A previous study (41) showed similar findings to those in the present study. Although the previous study showed that piribedil was associated with a higher incidence of AEs, piribedil exhibited a lower incidence of AEs in patients with early PD in the present study. This discrepancy may be related to the fact that we only analyzed early PD patients. The above-mentioned previous study analyzed early and advanced PD patients together and significant heterogeneity was observed. Rotigotine transdermal patch was more likely to cause headache and insomnia, as well as application site reactions, such as erythema and pruritus. Pramipexole IR/ER were more likely to cause insomnia.

Previous, we have performed a NMA to suggest ropinirole PR may be a better choice than other NEDAs as an adjunct to levodopa in advanced PD. Ropinirole is high efficacy agonists at D_2_ and D_3_ receptors, relevant to its neuroprotective properties. It has consistently been shown to reduce the risk of dyskinesia while controlling motor symptoms (4). Pulsatile stimulation of post-synaptic striatal dopamine receptors may be associated with motor complications. However, ropinirole PR, as a long-acting formulation, is provide continuous dopamine stimulation, which may improve dyskinesia of advanced PD (42). Therefore, Ropinirole PR may more suitable as an adjunct to levodopa for advanced PD. Piribedil is a unique profile of mixed D_2_/D_3_ receptor partial agonist and α-adrenoceptor antagonist properties. α2-adrenoceptor antagonism reinforces adrenergic, dopaminergic and cholinergic transmission to favorably influence motor function of dopaminergic neurons (43). And piribedil has a relatively short plasma half-life. Therefore, piribedil is beneficial in controlling symptoms of early PD in the relative absence of motor complications.

As with any NMA, some limitations should be mentioned to appropriately interpret the results of the present study. Firstly, the number of RCTs on some of the NEDAs was small, especially ropinirole PR and piribedil. Of the 20 RCTs included in this study, only one RCT evaluated ropinirole PR and two RCTs evaluated piribedil. Secondly, the RCTs included involved different trial designs. Of the studies included, five studies (27, 30, 32, 33, 35) were designed to show the noninferiority between the two formulations. No claims of superiority of one agent over another can be made based on the noninferiority study design of these studies (44). Therefore, further studies are also needed to confirm our findings. Finally, we only analyzed six commonly used NEDAs and subcutaneous apomorphine was not included in the present study.

## 5. Conclusions

The results of present NMA showed the six NEDAs were effective in early PD (except ropinirole PR in UPDRS-II). Compared with rotigotine transdermal patch, pramipexole IR/ER and ropinirole IR/PR, piribedil exhibited a better efficacy, especially as monotherapy. And ropinirole IR exhibited a higher incidence of AEs than other NEDAs. Importantly, our research may facilitate head-to-head research and larger sample sizes RCT to confirm the findings of this meta-analysis.

## Data availability statement

The original contributions presented in the study are included in the article/Supplementary material, further inquiries can be directed to the corresponding author.

## Author contributions

X-TC, QZ, and C-QZ participated in the study concept and design and prepared the manuscript. X-TC, QZ, F-FC, and S-YW participated in the acquisition, statistical analysis, and interpretation of the results. X-TC and C-QZ revised the draft paper for content. All authors contributed to the article and approved the submitted version.
