# Supplementary material for: Comparative efficacy and safety of six non-ergot dopamine-receptor agonists in early Parkinson's disease: a systematic review and network meta-analysis
Source: Front Neurol. 2023 Jun 16;14:1183823. doi: 10.3389/fneur.2023.1183823 (PMC10312085; doi:10.3389/fneur.2023.1183823)
Supplement: Supplementary file 1 [file Table_1.pdf]

Comparative Efficacy and Safety of Six Non-Ergot Dopamine-receptor Agonists in Early Parkinson's Disease: A Systematic Review and Network Meta-Analysis

Xiang-Ting Chen, Qian Zhang, Fei-Fei Chen, Si-Yuan Wen, Chang-Qing Zhou*

Department of Neurology, Bishan Hospital of Chongqing Medical University, Chongqing 402760, China

***Corresponding author**

Chang-Qing Zhou, MD, Chief Physician, Department of Neurology, Bishan Hospital of Chongqing Medical University, No. 9 Shuangxing Avenue, Bishan District, Chongqing, China 402760.

changqing_zhou@163.com

Appendix S1

Complete Search algorithm used in MEDLINE database

Disease

"Parkinson Disease"[MeSH Terms] OR "idiopathic parkinson s disease"[Tiab] OR "lewy body parkinson s disease"[Tiab] OR "parkinson s disease idiopathic"[Tiab] OR "parkinson s disease lewy body"[Tiab] OR "parkinson disease idiopathic"[Tiab] OR "parkinson s disease"[Tiab] OR "idiopathic parkinson disease"[Tiab] OR "lewy body parkinson disease"[Tiab] OR "primary parkinsonism"[Tiab] OR "parkinsonism primary"[Tiab] OR "paralysis agitans"[Tiab]

Treatments

"Dopamine Agonists"[Mesh] OR "DA"[tiab] OR "DAs"[tiab] OR "rotigotine" [Supplementary Concept] OR "rotigotine"[tiab] OR "ropinirole"[Supplementary Concept] OR "ropinirole"[tiab] OR "pramipexole"[Supplementary Concept] OR "pramipexole"[tiab] OR "piribedil"[Supplementary Concept] OR "piribedil"[tiab]

Design

((randomized controlled trial [pt] OR controlled clinical trial [pt] OR randomized controlled trials [mh] OR random allocation [mh] OR double-blind method [mh] OR single-blind method [mh] OR clinical trial [pt] OR clinical trials [mh] OR (“clinical trial” [tw]) OR ((singl* [tw] OR doubl* [tw] OR trebl* [tw] OR tripl* [tw]) AND (mask* [tw] OR blind* [tw])) OR (placebos [mh] OR placebo* [tw] OR random* [tw] OR research design [mh:noexp]) NOT (animals [mh] NOT human [mh])))

Complete Search algorithm used in EMBASE

'parkinson disease'/exp AND ('rotigotine'/exp OR 'ropinirole'/exp OR 'pramipexole'/exp OR 'piribedil'/exp OR 'dopamine receptor stimulating agent'/exp) AND 'randomized controlled trial'/exp

Complete Search algorithm used in the Cochrane Central Register of Controlled Trials

Parkinson’s disease [MeSH Terms]

Dopamine Agnoists

rotigotine transdermal patch [MeSH Terms]

ropinirole [MeSH Terms]

pramipexole [MeSH Terms]

piribedil [MeSH Terms]

#2 or #3 or #4 or #5 or #6

#1 or #7

randomized controlled trial [MeSH Terms]

#8 and #9

Appendix S2

Extracted Variables

The following study level variables were extracted:

Study design

RCTs;

Stratification type (age, gender, baseline measurements);

Allocation method;

Randomization method;

Blinding (single, double, open-label);

Primary and secondary endpoints;

Methods to account for missing data;

Study dates (database cut-off, primary analysis, secondary analysis);

Study phases;

Treatment duration;

Follow-up duration;

Inclusion and exclusion criteria;

Trial setting;

Trial location.

Intervention level variables:

Dosage;

Route of administration;

Frequency of administration;

Treatment period, inclusive of titration, dose reduction, and maintenance periods;

Concomitant therapies;

Background therapies;

Prior therapies.

Patient level variables:

Age;

Gender;

Ethnicity/race;

Hoehn and Yahr score;

UPDRS score (either II, III or both where available);

Patient reported questionnaire scores;

Outcome

Efficacy outcomes:

UPDRS-II: activities of daily living;

UPDRS-III: motor performance;

UPDRS-II+III

Tolerability outcomes

Overall withdrawals;

Withdrawals due to adverse events (AEs);

Withdrawals due to lack of efficacy.

Safety outcomes

the incidence of AE (≥ 1 AEs)

the incidence of serious AE (SAE)

AEs with a higher probability of occurrence

FIGURE S1 | Cochrane system bias evaluations for each of included publications.

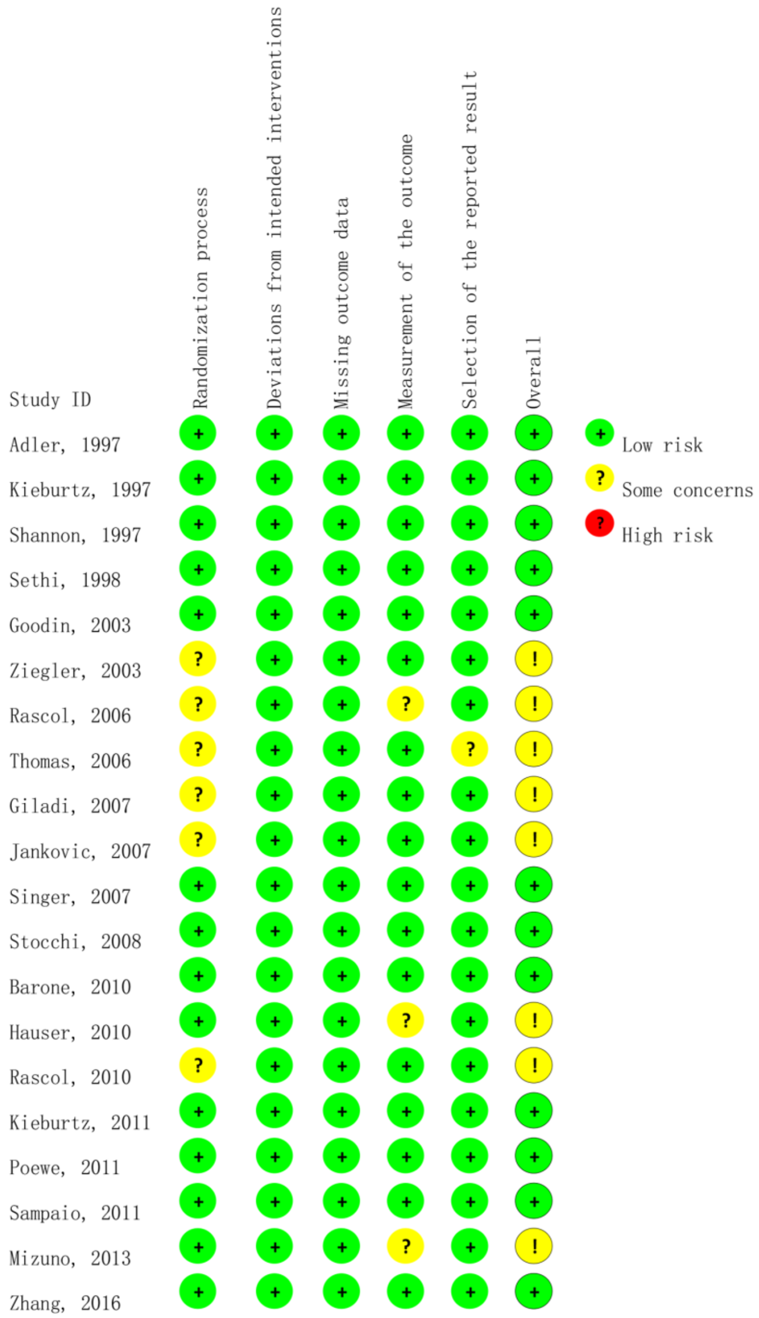

FIGURE S2 | Risk of bias graph.

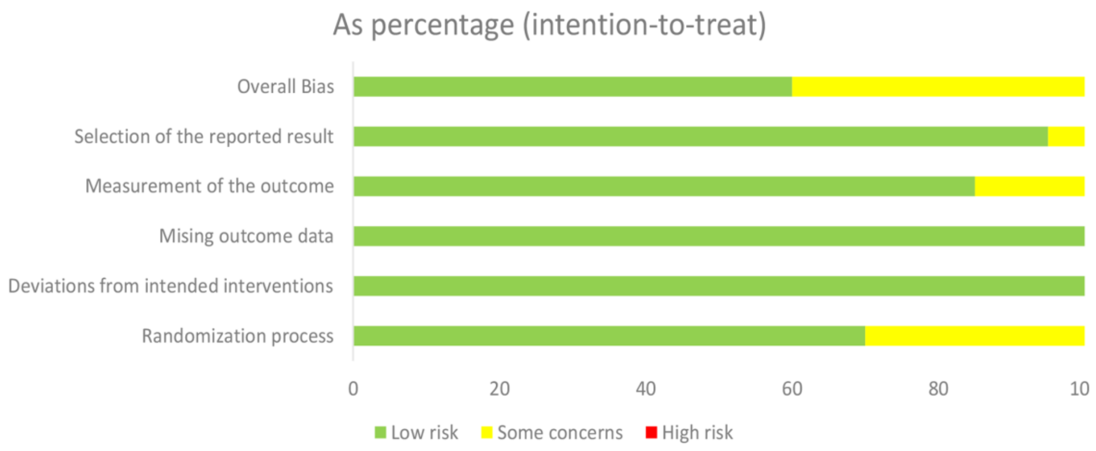

FIGURE S3 | Contribution plot of network meta-analysis.

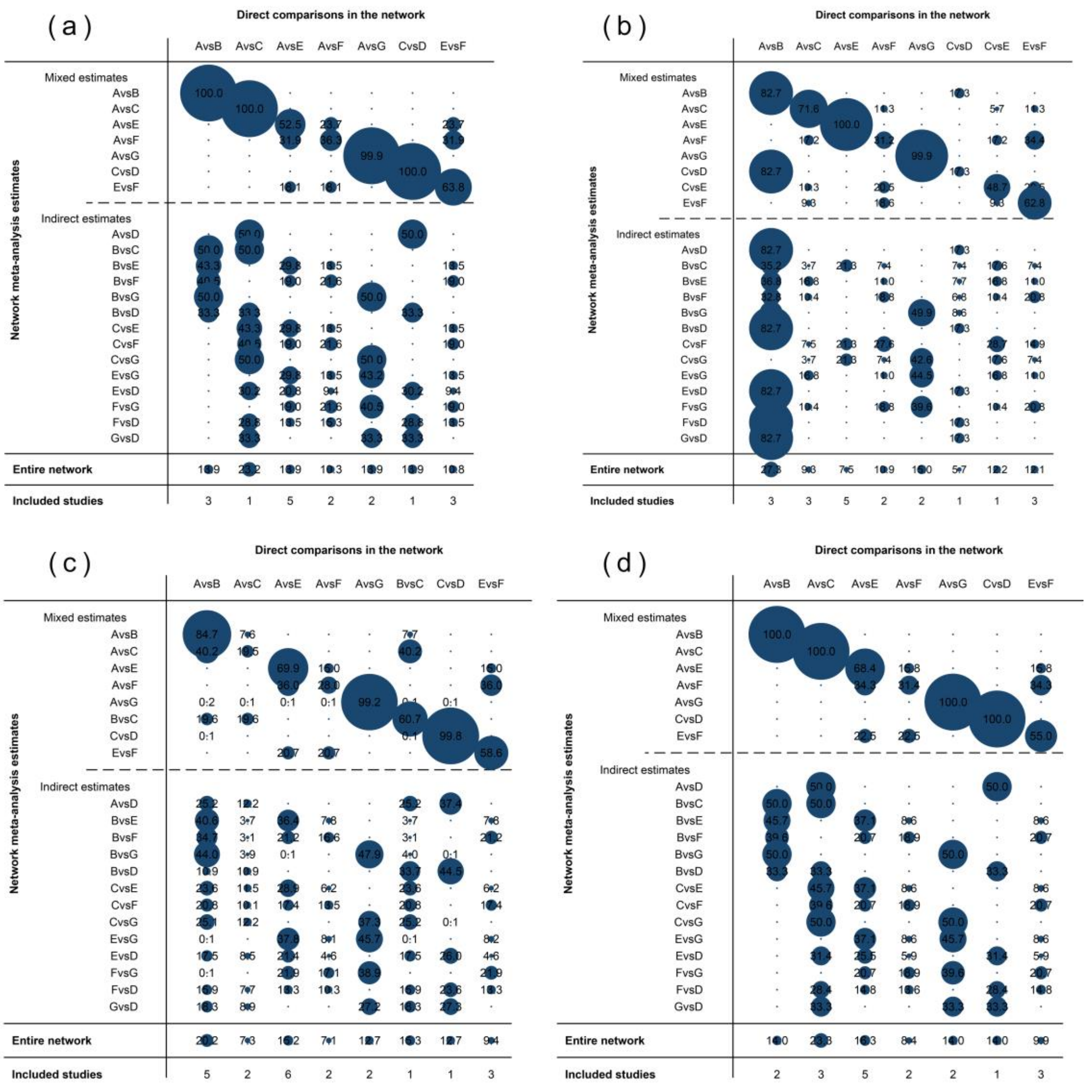

The size of each circle is proportional to the weight attached to each direct summary effect (horizontal axis) for the estimation of each network summary effects (vertical axis). The numbers re-express the weights as percentages. A: Placebo; B: Rotigotine transdermal patch; C: Ropinirole IR; D: Ropinirole PR; E: Pramipexole IR; F: Pramipexole ER; G: Piribedil. Note: (a) UPDRS-II, (b) UPDRS-III, (c) UPDRS-II+III, (d) ≥ 1 AEs.

TABLE S1 | Network meta-analysis results according to withdrawals by OR and 95% CrI.

	Placebo	Rotigotine transdermal patch	Ropinirole IR	Pramipexole IR	Pramipexole ER	Piribedil
**overall withdrawals (16studies, 4389patients, 19.71%)**						
Rank	5	4	6	2	**1**	3
SUCRA	0.241	0.374	0.037	0.716	**0.937**	0.695
Rotigotine transdermal patch	1.11 (0.79, 1.56)					
Ropinirole IR	0.80 (0.55, 1.16)	0.72 (0.47, 1.10)				
Pramipexole IR	**1.66 (1.18, 2.45)**	1.49 (0.91, 2.50)	**2.08 (1.27, 3.51)**			
Pramipexole ER	**2.18 (1.33, 3.70)**	**1.96 (1.08, 3.64)**	**2.73 (1.50, 5.13)**	1.32 (0.83, 2.02)		
Piribedil	1.60 (0.93, 2.76)	1.44 (0.76, 2.77)	**2.02 (1.03, 3.85)**	0.97 (0.49, 1.82)	0.73 (0.34, 1.55)	
**withdrawals due to AEs (19studies, 5206patients, 9.47%)**						
Rank	6	**1**	3	5	2	4
SUCRA	0.011	**0.681**	0.607	0.491	0.677	0.533
Rotigotine transdermal patch	**2.71 (1.45, 4.97)**					
Ropinirole IR	**2.50 (1.50, 4.11)**	0.92 (0.47, 1.88)				
Pramipexole IR	**2.23 (1.39, 4.06)**	0.83 (0.40, 1.98)	0.89 (0.47, 1.94)			
Pramipexole ER	**2.71 (1.25, 6.35)**	1.00 (0.38, 2.89)	1.08 (0.44, 2.92)	1.21 (0.57, 2.46)		
Piribedil	2.28 (0.89, 6.03)	0.84 (0.28, 2.66)	0.91 (0.32, 2.71)	1.02 (0.32, 2.92)	0.84 (0.23, 2.91)	
**withdrawals due to lack of efficacy (12studies, 3871patients, 5.30%)**						
Rank	**1**	2	5	6	4	3
SUCRA	**0.976**	0.553	0.293	0.270	0.431	0.476
Rotigotine transdermal patch	**0.36 (0.15, 0.86)**					
Ropinirole IR	**0.24 (0.12, 0.48)**	0.66 (0.25, 1.75)				
Pramipexole IR	**0.22 (0.08, 0.53)**	0.62 (0.16, 2.12)	0.94 (0.28, 2.89)			
Pramipexole ER	0.29 (0.07, 1.08)	0.82 (0.15, 3.85)	1.22 (0.26, 5.44)	1.33 (0.30, 5.51)		
Piribedil	0.33 (0.06, 1.43)	0.91 (0.13, 5.03)	1.39 (0.20, 6.93)	1.48 (0.20, 8.52)	1.11 (0.13, 8.41)	

Values in bold represent statistically significant results. The pooled incidence of AEs is indicated in parentheses. Bold font of SUCRA value indicates the highest SUCRA value, which can be interpreted as the estimated withdrawal proportion of higher than other treatments. OR: odds ratio; 95% CrI: 95% credibility interval; IR: immediate release; PR: prolonged release; ER: extended release; AEs: adverse events.

TABLE S2 | Network meta-analysis results according to AEs by OR and 95% CrI.

	Placebo	Rotigotine transdermal patch	Ropinirole IR	Ropinirole PR	Pramipexole IR	Pramipexole ER	Piribedil
**≥1 AEs (14studies, 3863patients,73.93%)**							
Rank	7	5	**1**	2	6	4	3
SUCRA	0.011	0.472	**0.847**	0.730	0.350	0.507	0.584
Rotigotine transdermal patch	**1.65 (1.02, 2.73)**						
Ropinirole IR	**2.41 (1.47, 4.29)**	1.48 (0.72, 3.08)					
Ropinirole PR	**2.24 (1.04, 4.99)**	1.36 (0.54, 3.42)	0.92 (0.52, 1.65)				
Pramipexole IR	**1.50 (1.09, 2.06)**	0.91 (0.50, 1.63)	0.62 (0.32, 1.13)	0.67 (0.28, 1.55)			
Pramipexole ER	**1.69 (1.10, 2.61)**	1.03 (0.53, 1.95)	0.70 (0.34, 1.39)	0.76 (0.30, 1.86)	1.13 (0.76, 1.64)		
Piribedil	**1.82 (1.17, 2.87)**	1.11 (0.56, 2.16)	0.75 (0.37, 1.48)	0.81 (0.33, 1.98)	1.21 (0.70, 2.13)	1.08 (0.58, 2.03)	
**SAE (10studies, 3087patients, 7.94%)**							
Rank	7	5	4	6	2	3	**1**
SUCRA	0.133	0.379	0.533	0.289	0.623	0.590	**0.953**
Rotigotine transdermal patch	1.40 (0.66, 3.19)						
Ropinirole IR	1.72 (0.83, 3.63)	1.23 (0.51, 2.74)					
Ropinirole PR	1.15 (0.27, 4.89)	0.82 (0.18, 3.71)	0.68 (0.19, 2.29)				
Pramipexole IR	**2.04 (1.05, 4.46)**	1.46 (0.52, 4.28)	1.19 (0.44, 3.58)	1.78 (0.37, 9.52)			
Pramipexole ER	2.06 (0.58, 7.64)	1.46 (0.32, 6.66)	1.20 (0.27, 5.56)	1.77 (0.26, 12.95)	1.00 (0.30, 3.27)		
Piribedil	**5.37 (1.88, 16.06)**	3.83 (1.01, 14.17)	3.13 (0.86, 11.67)	4.60 (0.76, 28.08)	2.64 (0.69, 8.95)	2.63 (0.47, 13.22)	

Values in bold represent statistically significant results. Bold font of SUCRA value indicates the highest SUCRA value, which can be interpreted as the estimated proportion of treatments worse than the treatment in question. The pooled incidence of AEs is indicated in parentheses.

TABLE S3 | AEs for six treatment modalities compared with placebo.

Treatment	Nausea (95% CrI)	Somnolence (95% CrI)	Dizziness (95% CrI)	Headache (95% CrI)	Fatigue (95% CrI)	Insomnia (95% CrI)	Constipation (95% CrI)
Rotigotine transdermal patch	**3.25 (1.82, 5.97)**	**2.48 (1.21, 5.40)**	1.61 (0.98, 2.63)	1.36 (0.73, 2.58)	2.92 (0.96, 11.49)	**2.04 (1.01, 4.31)**	1.34 (0.55, 3.42)
Ropinirole IR	**3.45 (1.72, 6.74)**	**3.73 (1.60, 9.05)**	**2.21 (1.34, 3.57)**	1.01 (0.48, 2.06)	**5.52 (1.24, 29.97)**	1.55 (0.72, 3.50)	1.7 (0.85, 3.42)
Ropinirole PR	3.30 (0.81, 13.03)	2.75 (0.47, 17.19)	2.35 (0.68, 8.03)	1.07 (0.26, 4.35)	2.67 (0.28, 28.06)	-	1.84 (0.41, 7.78)
Pramipexole IR	**2.63 (1.63, 4.55)**	**2.78 (1.52, 5.15)**	1.57 (0.99, 2.51)	0.99 (0.54, 1.74)	1.25 (0.67, 2.44)	**2.06 (1.11, 3.78)**	**3.64 (2.12, 6.92)**
Pramipexole ER	**2.59 (1.19, 6.03)**	**3.19 (1.24, 8.38)**	1.65 (0.74, 3.75)	0.74 (0.09, 6.93)	1.49 (0.43, 5.78)	-	**4.42 (2.11, 10.69)**
Piribedil	3.45 (0.91, 13.58)	2.21 (0.39, 12.76)	1.82 (0.70, 4.85)	-	-	2.35 (0.63, 9.47)	2.35 (0.71, 8.70)

Results are presented as ORs for safety events relative to interventions, with values greater than 1 indicating the comparator treatment is associated with higher odds of the AEs relative to interventions. Values in bold represent statistically significant results.

TABLE S4. Results of subgroup analyses among different time points.

(a) Subgroup analysis of UPDRS-II

UPDRS-II	Monotherapy (10studies, 3135patients)			Combined levodopa (3studies, 550patients)		
	MD (95% CI)	SUCRA	Rank	MD (95% CI)	SUCRA	Rank
Placebo	-	0.010	7	-	0.158	4
Rotigotine transdermal patch	**-1.61 (-2.36, -0.85)**	0.491	4	-	-	-
Ropinirole IR	**-1.53 (-2.79, -0.23)**	0.458	6	-	-	-
Ropinirole PR	-1.63 (-3.50, 0.25)	0.521	3	-	-	-
Pramipexole IR	**-1.80 (-2.45, -1.12)**	0.622	2	-1.21 (-2.87, 0.48)	0.689	2
Pramipexole ER	**-1.60 (-2.48, -0.71)**	0.476	5	-1.40 (-3.90, 1.15)	**0.738**	**1**
Piribedil	**-2.71 (-4.01, -1.39)**	**0.922**	**1**	-0.50 (-2.41, 1.45)	0.415	3

(b) Subgroup analysis of UPDRS-III

UPDRS-III	Monotherapy (11studies, 3187patients)			Combined levodopa (5studies, 1032patients)		
	MD (95% CI)	SUCRA	Rank	MD (95% CI)	SUCRA	Rank
Placebo	-	0.000	7	-	0.113	5
Rotigotine transdermal patch	**-3.4 (-4.71, -2.04)**	0.245	6	-	-	-
Ropinirole IR	**-4.31 (-6.18, -2.43)**	0.441	5	**-5.51 (-10.15, -0.70)**	**0.827**	**1**
Ropinirole PR	**-5.83 (-8.99, -2.68)**	0.756	2	-	-	-
Pramipexole IR	**-4.81 (-5.95, -3.6)**	0.596	3	-2.19 (-8.56, 4.21)	0.400	4
Pramipexole ER	**-4.52 (-6.10, -2.83)**	0.503	4	-3.71 (-12.76, 5.8)	0.606	2
Piribedil	**-7.51 (-9.94, -5.14)**	**0.960**	**1**	-3.33 (-10.48, 3.81)	0.554	3

(c) Subgroup analysis of UPDRS-II+III

UPDRS-II+III	Monotherapy (13studies, 3867patients)			Combined levodopa (3studies, 550patients)		
	MD (95% CI)	SUCRA	Rank	MD (95% CI)	SUCRA	Rank
Placebo	-	0.000	7	-	0.103	4
Rotigotine transdermal patch	**-4.27 (-5.64, -2.99)**	0.220	6	-	-	-
Ropinirole IR	**-7.21 (-9.04, -5.36)**	0.681	3	-	-	-
Ropinirole PR	**-8.80 (-12.61, -4.98)**	0.831	2	-	-	-
Pramipexole IR	**-5.30 (-6.70, -3.88)**	0.401	5	-3.38 (-8.57, 1.83)	0.573	3
Pramipexole ER	**-5.44 (-7.52, -3.43)**	0.427	4	-4.50 (-12.35, 3.29)	**0.702**	**1**
Piribedil	**-10.20 (-13.51, -6.86)**	**0.941**	**1**	-3.82 (-10.49, 2.83)	0.622	2

(d) Subgroup analysis of overall withdrawals

overall withdrawals	Monotherapy (13studies, 3822patients)			Combined levodopa (3studies, 567patients)		
	OR (95% CI)	SUCRA	Rank	OR (95% CI)	SUCRA	Rank
Placebo	-	0.237	5	-	0.456	3
Rotigotine transdermal patch	1.11 (0.79, 1.57)	0.362	4	-	-	-
Ropinirole IR	0.80 (0.55, 1.18)	0.041	6	-	-	-
Pramipexole IR	1.84 (1.23, 2.87)	0.712	3	1.13 (0.48, 2.69)	0.589	2

Pramipexole ER	**2.50 (1.47, 4.41)**	**0.932**	**1**	0.76 (0.12, 5.26)	0.343	4
Piribedil	1.83 (0.92, 3.60)	0.716	2	1.20 (0.42, 3.49)	**0.612**	**1**

(e) Subgroup analysis of ≥1 AEs

⩾1 AEs	Monotherapy (9studies, 2814patients)			Combined levodopa (5studies, 1032patients)		
	OR (95% CI)	SUCRA	Rank	OR (95% CI)	SUCRA	Rank
Placebo	-	0.045	7	-	0.130	5
Rotigotine transdermal patch	1.68 (0.92, 3.26)	0.511	3	-	-	-
Ropinirole IR	**2.47 (1.04, 6.09)**	**0.795**	**1**	2.43 (0.93, 6.78)	**0.742**	**1**
Ropinirole PR	2.26 (0.70, 7.51)	0.693	2	-	-	-
Pramipexole IR	1.58 (0.99, 2.52)	0.450	6	1.36 (0.49, 3.91)	0.370	4
Pramipexole ER	1.65 (0.90, 3.00)	0.495	5	1.78 (0.38, 8.51)	0.562	3
Piribedil	1.69 (0.77, 3.61)	0.511	4	2.27 (0.71, 7.33)	0.695	2

Values in bold represent statistically significant results.

FIGURE S4 | Results of the node splitting method.

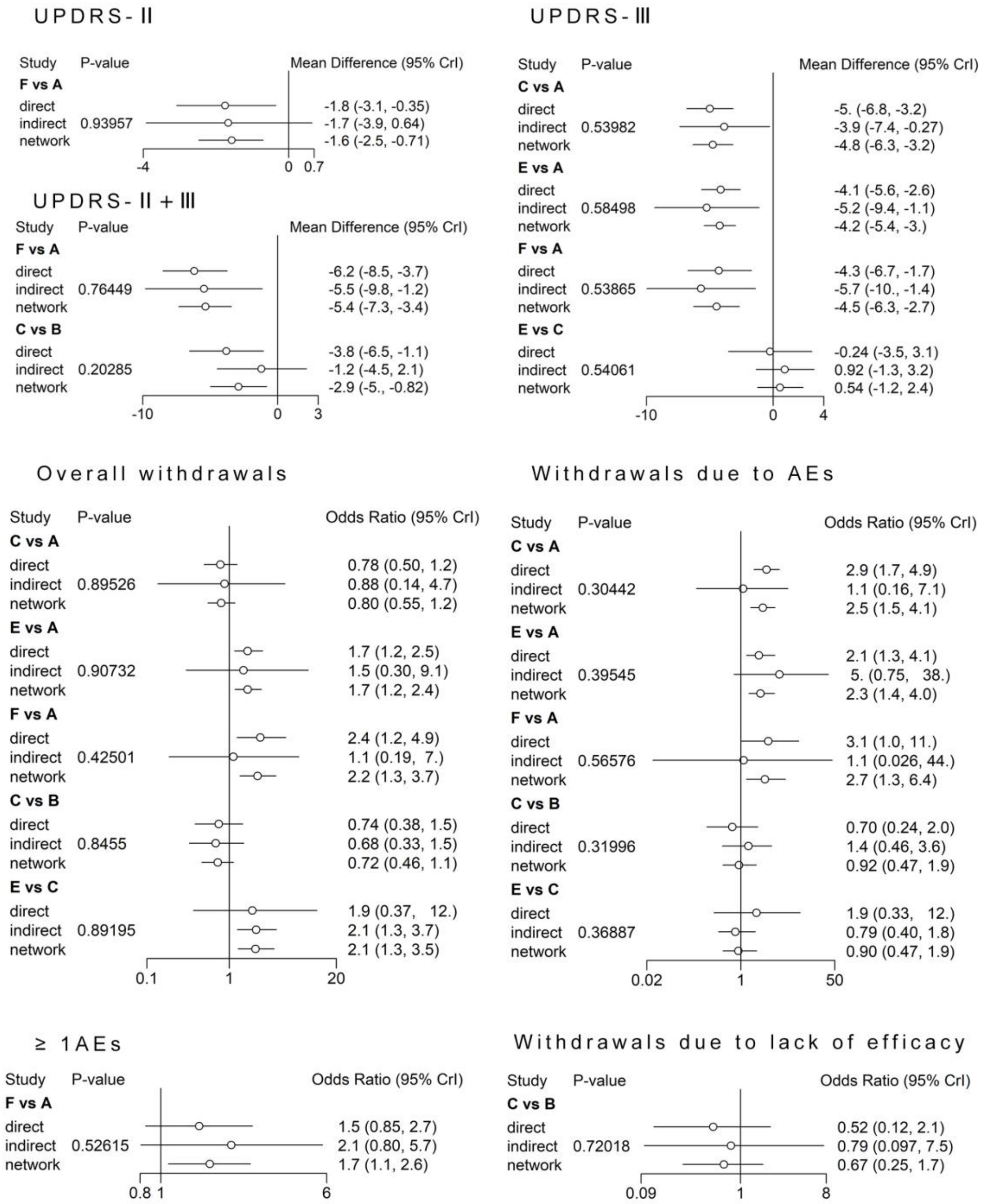

P-value means the consistency of direct and indirect evidence, p>0.05 means there is no significant inconsistency. A: Placebo; B: Rotigotine transdermal patch; C: Ropinirole IR; D: Ropinirole PR; E: Pramipexole IR; F: Pramipexole ER; G: Piribedil.

FIGURE S5 | Net heat plot to assess inconsistency.

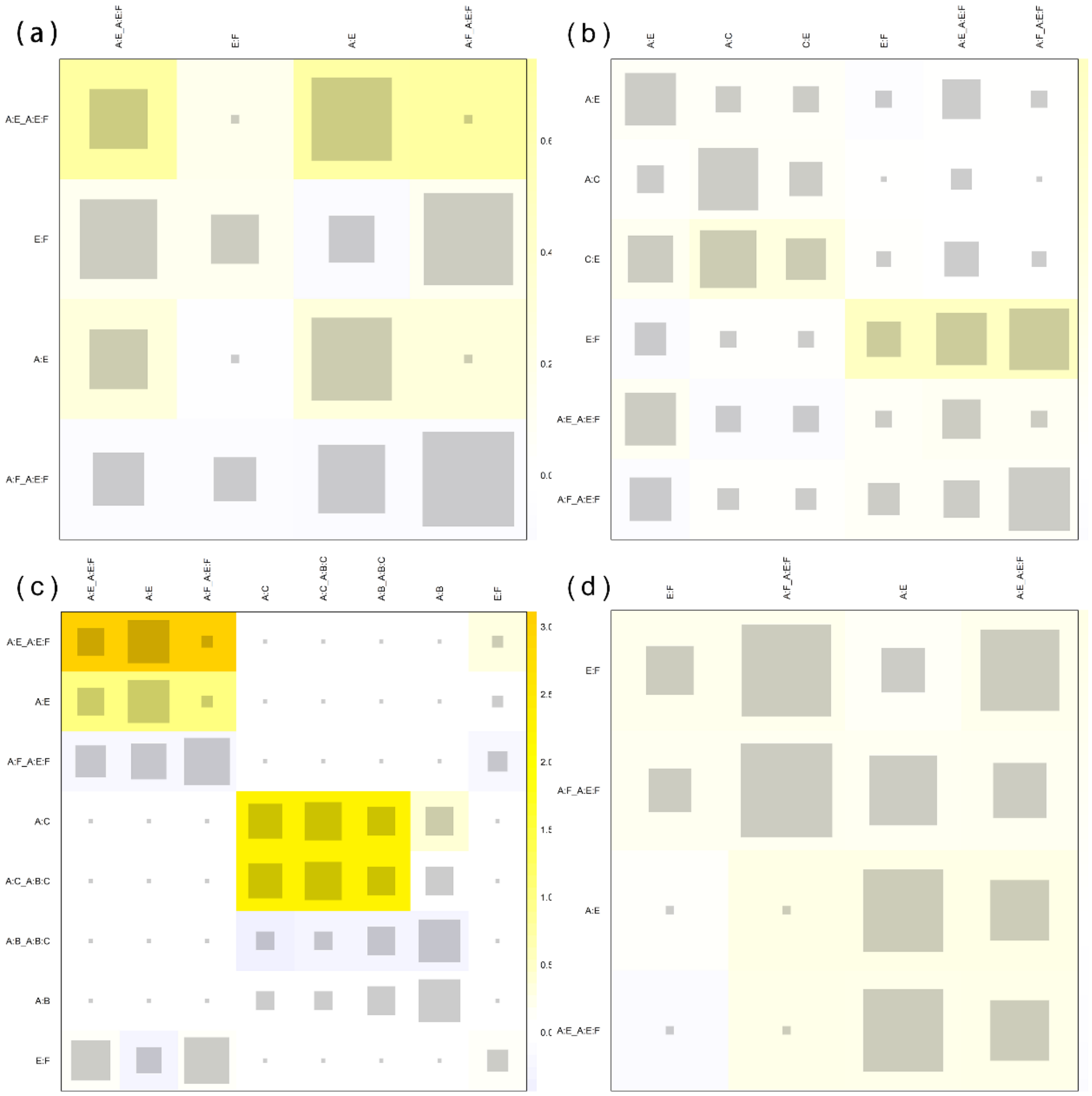

The size of the gray squares indicates the contribution of the direct evidence (shown in the column) to the network evidence (shown in the row). The colors are associated with the change in inconsistency between direct and network evidence. A blue-colored element indicates that the evidence of the design in the column supports the evidence in the row; a warm-colored element indicates that significant inconsistency may arise from a specific design or comparison, and this trend is illustrated by the intensity of the color. A: Placebo; B: Rotigotine transdermal patch; C: Ropinirole IR; D: Ropinirole PR; E: Pramipexole IR; F: Pramipexole ER; G: Piribedil. Note: (a) UPDRS-II, (b) UPDRS-III, (c) UPDRS-II+III, (d) ≥ 1 AEs.

FIGURE S6 | Comparison-adjusted funnel plots for assessment of publication bias.

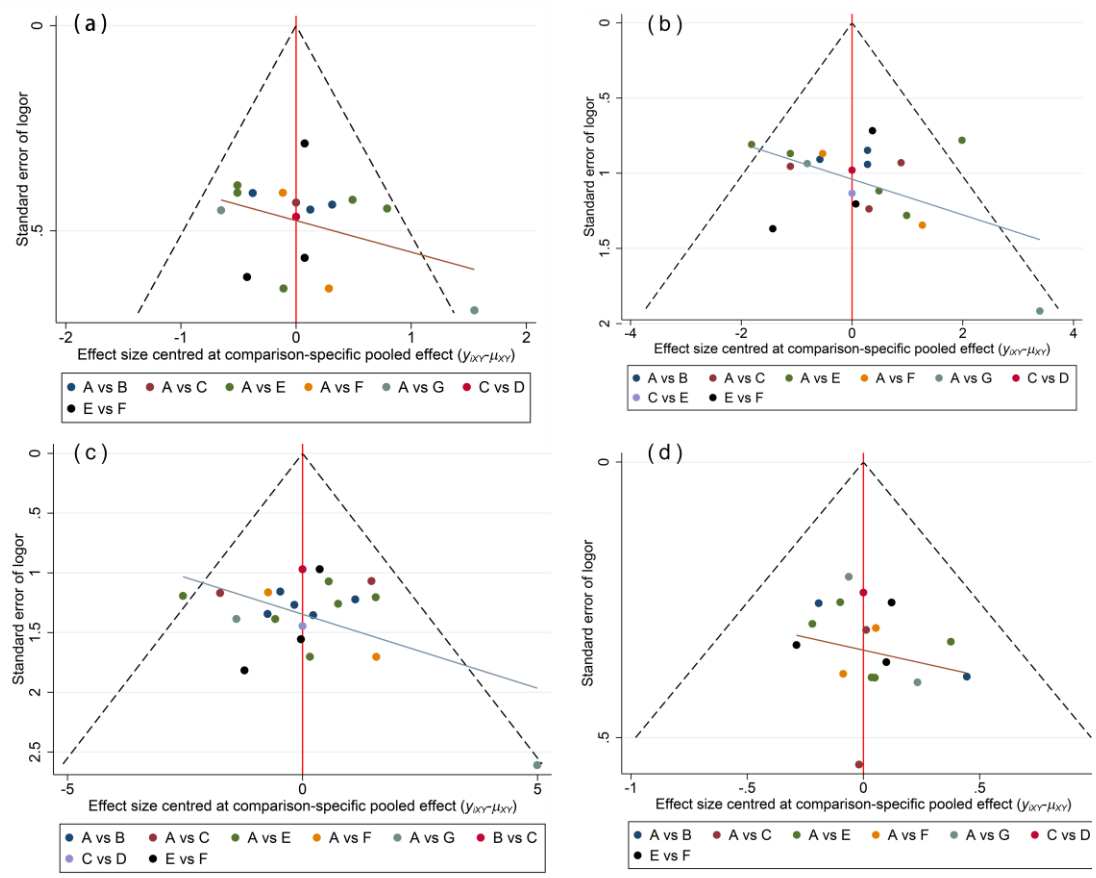

The horizontal axis represents an adjusted effect size, presenting the difference between each study's estimate (yiXY) from the direct summary effect for each comparison (yiXY −µXY), and the vertical axis represents the SE of yiXY. The dashed lines represent pseudo 95% confidence intervals. Note: (a) UPDRS-II, (b) UPDRS-III, (c) UPDRS-II+III, (d) ≥ 1 AEs.
